# Draft Genome Sequence of the Plant Growth-Promoting Rhizobacterium Pseudomonas protegens Strain BNJ-SS-45, Isolated from Rhizosphere Soil of Wheat (Triticum aestivum)

**DOI:** 10.1128/MRA.00926-18

**Published:** 2018-08-30

**Authors:** Apekcha Bajpai, Kishor K. Shende, Narendra Meena, Prashanth Suravajhala, Krishna Mohan Medicherla, Bhavdish Narain Johri

**Affiliations:** a Department of Biotechnology, Barkatullah University, Bhopal, MP, India; b Department of Biotechnology and Bioinformatics, Birla Institute of Scientific Research, Jaipur, RJ, India; Queens College

## Abstract

Here, we present the draft genome sequence of Pseudomonas protegens strain BNJ-SS-45, which was isolated from wheat rhizosphere. The genome is assembled with 7,116,445 bp with a GC content of 63.34% consisting of 32 scaffolds. The genome is useful in prediction of secondary metabolites, particularly rhizoxin, pyoverdine, and bacteriocin.

## Abstract (precis)

Here, we present the draft genome sequence of Pseudomonas protegens strain BNJ-SS-45, which was isolated from wheat rhizosphere. The genome is assembled with 7,116,445 bp with a GC content of 63.34% consisting of 32 scaffolds.

## ANNOUNCEMENT

Pseudomonas protegens strain BNJ-SS-45 is a rhizospheric bacterium isolated from wheat. This strain has been previously examined for its plant growth-promoting attributes and prediction of secondary metabolites, such as antifungals, biosurfactants, and siderophores, and is classified as a plant growth-promoting rhizobacterium (PGPR) (1). Pure culture cells were grown in 50 ml Kings B broth (KB) in a 250-ml shake flask overnight at 30°C, and genomic DNA was isolated using Ausubel et al.’s protocol (2). The concentration obtained was 569.6 ng/μg, and the *A*_260/280_ value was 1.9. A draft genome sequence of strain BNJ-SS-45 was generated from a paired-end library constructed with the Nextera library preparation kit. The sequencing was performed on an Illumina HiSeq 2500 device which generated 8,076,066 reads (estimated insert size, ∼350 bp). Reads were processed with the A5-MiSeq pipeline (version 20160825), following which adapter trimming, quality filtering, error correction, and contig and scaffold generation were carried out (3). High-quality reads were *de novo* assembled with the A5-MiSeq pipeline into scaffolds with a minimum length of 650 bp, ranging from 673 bp to 1,426,290 bp, and read length of *N*_50_ of 828,551. The assembly was cross-checked against another *de novo* assembler, *viz.*, Velvet (4), resulting in 79 contigs with a GC content of 63.34%. Gene prediction was performed using the prokaryotic gene finder tool Glimmer (5) with “archaea bacterial generic” as the closest organism. The prediction resulted in 6,357 candidate protein-coding genes, 150 repeat regions, 61 tRNAs, and 6 rRNAs.

Functional annotation was performed using BLAST search (E value, 10^−5^) to infer the predicted protein sequences against the UniProt Knowledgebase (UniProtKB) (6). Gene model descriptions were refined using InterProScan (7) for the identification of conserved protein domains and gene ontology (GO) term retrieval with the KEGG Automatic Annotation Server (KAAS) (8) for KEGG Orthology (KO) assignments and pathway mapping. When the genome was queried using antiSMASH (9), a genome-wide analysis tool for inferring secondary metabolites, we obtained the clusters related to rhizoxin and pyoverdine gene clusters and bacteriocin, among others. From the antiSMASH results, we observed significantly enriched gene-encoding enzymes involved in siderophore biosynthesis and a gene-encoding enzyme for nonribosomal protein sequences, in addition to enzymes involved in biosynthesis of polyketides and nonribosomal peptides associated with biocontrol activity against phytopathogens. Further full annotation and assessment of enzymes for this strain would provide insights into the versatility of this genome as a PGPR.

### Data availability.

This whole-genome shotgun project has been deposited in GenBank with the accession number PYJM00000000 under the BioProject number PRJNA435479.
